# Refitting and Repairing Rubber Plates

**Published:** 1912-11-15

**Authors:** J. L. Mewburn


### REFITTING AND REPAIRING RUBBER PLATES.

BY DR. J. L. MEWBURN.

Read before Tennessee State Dental Society, June 7, 1912.

The substance of this paper was published as an open letter in the May number of the *Dental Brief*, but was so condensed and abbreviated, judging from a number of letters which I have received asking for more explicit details, that I must have muddled things somewhat, so I concluded to rewrite it and present it as a contribution to this meeting.

On page 170, March number of the Dental Brief, Dr. J. Oscar Hall, of Waco, Texas, in an illustrated article describes what he claims to be "an easy, accurate, quick and new method of re-making an upper denture."

I recognize that we have a great profession, and, I am keenly interested in its general welfare and progress. I also recognize that every section of our country has furnished men who have brought honor to us because, as members of the dental profession they are conspicuous. As a citizen of such a country and a member of such a profession, I am interested in all that is *true* advancement and hail its coming, regardless of its source, but I consider it my duty and pleasure to guard with zealous care all the inherent rights in such achievements which pioneer members of our profession have handed down to us.

In the light of recent scientific advancement, therefore, and for the benefit and information of the young men in our profession, my 47 years of practical experience emboldens me to say that Dr. Hall's whole procedure should now be considered impracticable and imperfect, if not obsolete.

As a working principle I would say that in all cases where it is necessary to make additions to, or to mend broken plates, upper or lower, or to bush or refit an old plate with new rubber, it is absolutely necessary to obtain *perfect* union, not mechanical adaptation, of the new rubber to the old, or the work is a failure.

In my experiences this can be done best in the following manner:—First provide a supply of red and pink vulcanizable gutta-percha; a pound can of carbon di-sulfid; a heavy, double end spatula, one end curved the other straight; a short wide mouthed bottle, which should be kept one-third full of the red gutta-percha, cut into small pieces and dissolved to a creamy consistency by the addition of carbon di-sulfid; and a small stump bristle brush. The vulcanizable gutta-percha is made by the S. S. White Co., and the Eugene Doherty Co., the waxable gutta-percha by Hood. I prefer Hood's. The ordinary rubber will not answer for this work.

When the rubber solution becomes too thick for use, from evaporation of the carbon di-sulfid, add a little more of the di-sulfid and set the bottle in a vessel of hot water, stopper removed, shake or stir occasionally, which will soon give the proper thick creamy consistence.

When an old plate becomes a misfit from alveolar absorption, take a new impression, and run a plaster model an inch thick and while this is hardening dip the old plate in the pickeling jar of a 25% solution of sulphuric acid, then with pumice on a stiff brush wheel and the laboratory lathe, give the whole piece, teeth and all, a thorough scrubbing; wash, dry and with a large round or pear shaped cross-cut bur in the dental engine, freshen and slightly roughen the entire palatine surface of the plate; paint this over with the rubber solution, over which press with the fingers a sheet of the red gutta-percha, softened in hot water and with scissors trim the surplus from around margin of plate.

Before reaching this stage however, see that the plaster model is hard, and has had its surface rubbed with talcum on a bunch of cotton to keep the gutta-percha from sticking, also that the model has been on your heating apparatus and gradually warmed up until just now it is so warm that you can scarcely bear your hand on it. Remove the hot model from heater and with fingers and thumbs press the plate with gutta-percha lining upon it until the softened gutta-percha begins to squeeze out from under the edges of the plate. Now, transfer the plate with gutta-percha lining softened with as much heat as the patient can bear in the mouth while biting to a correct occlusion. To open the bite, add another sheet of gutta-percha, and a third if necessary, proceed as above, reheating again and again if necessary, changing it from the mouth to the model and back again until a perfect impression and occlusion are obtained; then transfer to the model and flask or leave off the model and flask the piece.

In either case make but one investment, close down the bolts, and as soon as the plaster is hard vulcanize and finish.

No retaining points, nor countersunk holes are necessary. The rubber solution seems to act like a flux in soldering metals and you will find the old and new rubbers in absolutely perfect union when the vulcanizing is done.

In the article referred to, the author says:—"If you want to open the bite a fraction it can be done by using very thin wax in the plate and not closing the flask entirely. Screw the heel bolt a fraction closer than the others or the molar teeth will have a tendency to strike first. Faults in occlusion may oftentimes be bettered, but I would not advise any one to try it until entirely familiar with the *Modus,, Operandi.*" I feel prompted to strongly criticise any such methods, because of their faulty technique which will surely produce inacurate and unsatisfactory repairs. We can not expect to raise the bite by partially closing a flask, as the vulcanite flows readily when heated to the vulcanizing point, and the flasks will close entirely or unequally.

For all repair work there is nothing equal to this vulcanizable gutta-percha, the di-sulfid solution, and the hot spatulation. Cut into strips and squares a quantity of the red and pink gutta-percha for the case in hand, and have the parts where the new rubber is to be packed onto the old rubber, freshened and roughened and painted with the rubber solution. Most of these mending pieces may be packed in the hand, and several may be vulcanized at the same time by placing them in layers as you fill the flask with plaster.

New full lower cases may be completed in one third the time required by the old wax and opened flask method by the following process: After the bite has been taken and the models are set in the articulator, remove the base plate and bite wax from the lower model, then paint the model with the rubber solution, and with the hot spatula, you can mold or spread on the red and pink gutta-percha around each tooth as you place it in artistic contour as rapidly and as beautifully as you please. The whole piece can then be invested solidly in the flask and vulcanized without opening the flask insuring correct occlusion and saving time.

I have been using the vulcanizable gutta-percha about 8 or 10 years, but the carbon di-sulfid solution of rubber I picked up from one of the journals about 3 years ago. This I call "rubber flux" or the cement substance which binds, inseparably the new rubber to the old.

I claim no originality for any part of this method. I have only written up the technique of the process, so that others may be benefited as much as I have been.

### DISCUSSION.

Dr. Holder. The suggestion made by Dr. Mewburn in regard to soluable vulcanite, I have found very helpful indeed. Repairing old vulcanite plates I have never found very practical or satisfactory. The fact of the matter is that I have never found one of those cases, if it needed to be refitted where I thought that a mere patch could be made that was adequate. I do not believe that one plate out of fifty, no not one out of one hundred so repaired will occlude properly. You may think that is a little strange, and a serious accusation to be brought against the usual practice of dentists for these conditions, but if you investigate the subject carefully you will not find an articulation that will properly occlude after the repair is made. I have been caught in the matter myself. We may articulate the teeth so they will open and shut, but they will not occlude so that the patient can use them unconsciously.

I believe that this question of the proper articulation of the teeth is one that is going to attract a great deal more attention in the next few years than it has in the past. I must confess that I do not like laboratory work as well as some people do. I prefer to work at the chair, and therefore, see a great deal of poor prosthetic work breaking down; but where I have to make a set of teeth, I try to do the very best I possibly can. I want to do it the best way and want the articulation certain, so that the teeth are well articulated, you simply can not do this by the old method.

Dr. J. M. Cole. The talk put forth in the paper is timely and should be profitable. I have used the first method for replacing new rubber in old plates that he referred to in my practice in Dyersburg, and have told one or two of my confreres that it was a good method, but it has been a complete failure in many cases. The vulcanizable gutta-percha and refitting it on the old plate with the flux solution, I believe is bound to be a success. I have made two attempts recently in refitting sets of teeth by the old method and I have made a failure in both cases. I am certainly going to investigate this method a little more closely, as soon as I get home. I am going to give this new method a trial and I am satisfied that I shall be successful.

DR. J. L. MEWBURN, in closing. I have nothing further to offer, except that I want it understood that this paper is only a criticism of the paper from the Texas Dentist who published his method and claimed it as new. I just want to show the young men in our Association that it was an old and very unreliable procedure. I have refitted old vulcanite plates frequently by the method so generally mentioned. I have taken the entire old rubber out and put new rubbei back and then fallen down on the operation. We must charge less for a repair than for a new plate, but we have patients sometimes that will not have a new plate, even though we know they would be better off with one, and all these things have to be considered. You have to look at both sides of the question.

